# Knockout of Akt1/2 suppresses the metastasis of human prostate cancer cells CWR22rv1 in vitro and in vivo

**DOI:** 10.1111/jcmm.16246

**Published:** 2020-12-29

**Authors:** Bing Su, Lijuan Zhang, Wenfang Zhuang, Wei Zhang, Xiaofan Chen

**Affiliations:** ^1^ Medical laboratory Shidong Hospital Affiliated to University of Shanghai for Science and Technology Shanghai China; ^2^ Department of Laboratory Medicine The Third Affiliated Hospital of Xinxiang Medical University Xinxiang China; ^3^ Biomedical Research Institute Shenzhen Peking University‐the Hong Kong University of Science and Technology Medical Center Shenzhen China

**Keywords:** Akt, AR, FOXO, metastasis, prostate cancer

## Abstract

Although primary androgen deprivation therapy resulted in tumour regression, unfortunately, majority of prostate cancer progress to a lethal castration‐resistant prostate cancer, finally die to metastasis. The mutual feedback between AKT and AR pathways plays a vital role in the progression and metastasis of prostate cancer. Therefore, the treatment of a single factor will eventually inevitably lead to failure. Therefore, better understanding of the molecular mechanisms underlying metastasis is critical to the development of new and more effective therapeutic agents. In this study, we created prostate cancer CWR22rv1 cells with the double knockout of Akt1 and Akt2 genes through CRISPR/Cas9 method to investigate the effect of Akt in metastasis of prostate cancer. It was found that knockout of Akt1/2 resulted in markedly reduced metastasis in vitro and in vivo, and appeared to interfere AR nuclear translocation through regulating downstream regulatory factor, FOXO proteins. It suggests that some downstream regulatory factors in the AKT and AR interaction network play a vital role in prostate cancer metastasis and are potential targeting molecules for prostate cancer metastasis treatment.

## 1 INTRODUCTION

Prostate cancer (CaP) is the most common male malignant tumour in western societies, accounting for the second place in male cancer mortality.^1^ Despite the obvious early effects of androgen deprivation therapy, most patients inevitably develop castration‐resistant prostate cancer (CRPC) and eventually die of metastasis—the leading cause of CaP death.^2^ Due to the heterogeneity of CaP and poor overlap between specimens, it is extremely difficult to find common targets, resulting in no effective treatment currently. Therefore, there is an urgent need to elucidate the mechanism of CaP development and progression, in order to develop new and effective therapeutic approaches.

Our previous research used a shRNA library targeting the human genome to screen for CaP metastasis‐related genes and identified forkhead box protein O 4 (FOXO4) and gamma‐aminobutyric acid (GABA) a receptor‐associated protein‐like 1 (GABARAPL1), two new CaP invasion and metastasis inhibitory genes.^3^, ^4^ Interestingly, the two genes converged on the Akt (also known as protein kinase B, PKB) signalling pathway, which is a major regulator of CaP metastasis.^5^ For example, the mutations in genes such as PI3K, EGFR and HER2 cause Akt to be highly activated, and then, Akt regulates its downstream signalling pathways or through mutual feedback with other signalling pathways to promote tumorigenesis and progression.^6^

The Akt family includes three members (Akt1, Akt2 and Akt3), of which Akt1/2 has been recognized as an oncogene, but the function of Akt3 is unclear. The knockout studies of mouse embryos have shown that simultaneous knockout of Akt1 and Akt2 can cause embryo death. Although Akt1 knockout alone does not kill the embryo, it suppresses tumorigenesis in Pten ± mice.^7^ Because most tumours occur in adulthood, in order to better study the role of Akt in tumours, Hay Labs studied the effect of Akt knockout on p53−/− adult mouse lymphoma and found that Akt knockout slowed down tumour progression.^8^ These embryonic and adult mouse studies have shown that Akt is necessary for basic life functions and plays a vital role in tumorigenesis and development.

Although previous studies carried out in various species and cell types have provided the foundation for our current understanding of the Akt and its associated signal pathway network, the functions and mediators that actually act to control CaP development and progression are not yet well defined. At present, the CRISPR/Cas9 method has rapidly swept the entire scientific research field and has become a revolutionary technology for DNA mutation and editing. In this study, we knockout Akt1 and Akt2 genes in CaP CWR22rv1 cells to explore the importance of Akt in CaP metastasis and its regulated downstream factors in order to find specific targets.

## 2 MATERIAL AND METHOD

### 2.1 Cell culture

Human prostate cancer cell lines of CWR22Rv1 were purchased from the Chinese Academy of Cell Bank. CWR22Rv1 cells were cultured in RPMI 1640 media supplemented with 10% FBS and incubated at 37°C in a humidified incubator containing 5% CO_2_.

### 2.2 CRISPR/Cas9 design and lentivirus transfection

Use software to design hAKT‐gRNA sequences. hAKT1‐gRNA‐F1:CACCGAAGGTGCGTTCGATGACAG and hAKT1‐gRNA‐R1:AAACCTGTCATCGAA CGCACCTTC; hAKT2‐gRNA‐F1: CACCGTCTCGTCTGGAGAATCCACG and hAKT2‐gRNA‐R1: AAACCGTGGATTCTCCAGACGAGAC, and then insert into the px300 plasmid to construct the px300‐HAkt‐gRNA plasmid. The CaP CWR22rv1 cells were simultaneously transfected with px330‐AKT1, px330‐AKT2 and plko plasmid with puromycin tag, then puromycin screened, and monoclonal cells were isolated and sequenced.

### 2.3 Primary tumour growth

3‐week‐old BALB/c nude mice (Beijing Vital River Laboratory Animal Technology Co., Ltd.) were injected s.c. into the flank regions with 2 × 10^6^ Akt1 and Akt2 knockout (marked as Akt1/2 DKO) CWR22rv1 cells mixed Matrigel. The viability of the cells was >90% as determined by trypan blue exclusion. Tumour volume (cubic millimetres) was measured using a calliper, applying the formula [volume = length × width × width/2] for approximating the volume of a spheroid. Tumour burden per mouse was calculated by accumulating the tumour volume thrice weekly. After the tumour volume is >4 cm^3^, the animals were killed; lymph node and lungs were examined for visible metastatic foci after H&E staining. All animal protocols were approved by the Institutional Animal Care and Use Committee of Shenzhen Biochemical Institute.

### 2.4 Immunoblot analysis

Cell lysates were generated, and Western blotting with Akt1, Akt2, FOXO1, FOXO3, FOXO4, AktpT308, FOXOpT24/32 and GAPDH antibodies were carried out as described previously.^9^

### 2.5 Invasion assay

Modified Matrigel‐based Boyden chamber assays were performed as previously described.^4^

### 2.6 Quantitative reverse transcriptase PCR (qRT‐PCR)

Total RNA was isolated using TRIzol Reagent (Sigma) and was reverse transcripted into cDNA using Revert Aid First Strand cDNA Synthesis Kit (Thermo Scientific). Real‐time PCR was performed using SYBR premix EX Taq (TaKaRa) and analysed with CFX96 Real‐Time System (Bio‐Rad). Real‐time primer sequences were listed in Table S1. GAPDH was used as a housekeeping gene for the qRT‐PCRs. Each test was done in triple replication, and the 2^−ΔCt^ method was used to calculate the expression of genes.

### 2.7 Statistical analyses

Statistical significances between groups were determined by two‐tailed Student's *t* test. All statistical analyses were performed by using SPSS 16.0 software program. *P* < .05 was considered statistically significant.

## 3 RESULTS

### 3.1 Effects of Akt1/2 knockout on metastasis in CaP CWR22rv1 cells in vitro

**FIGURE 1:**
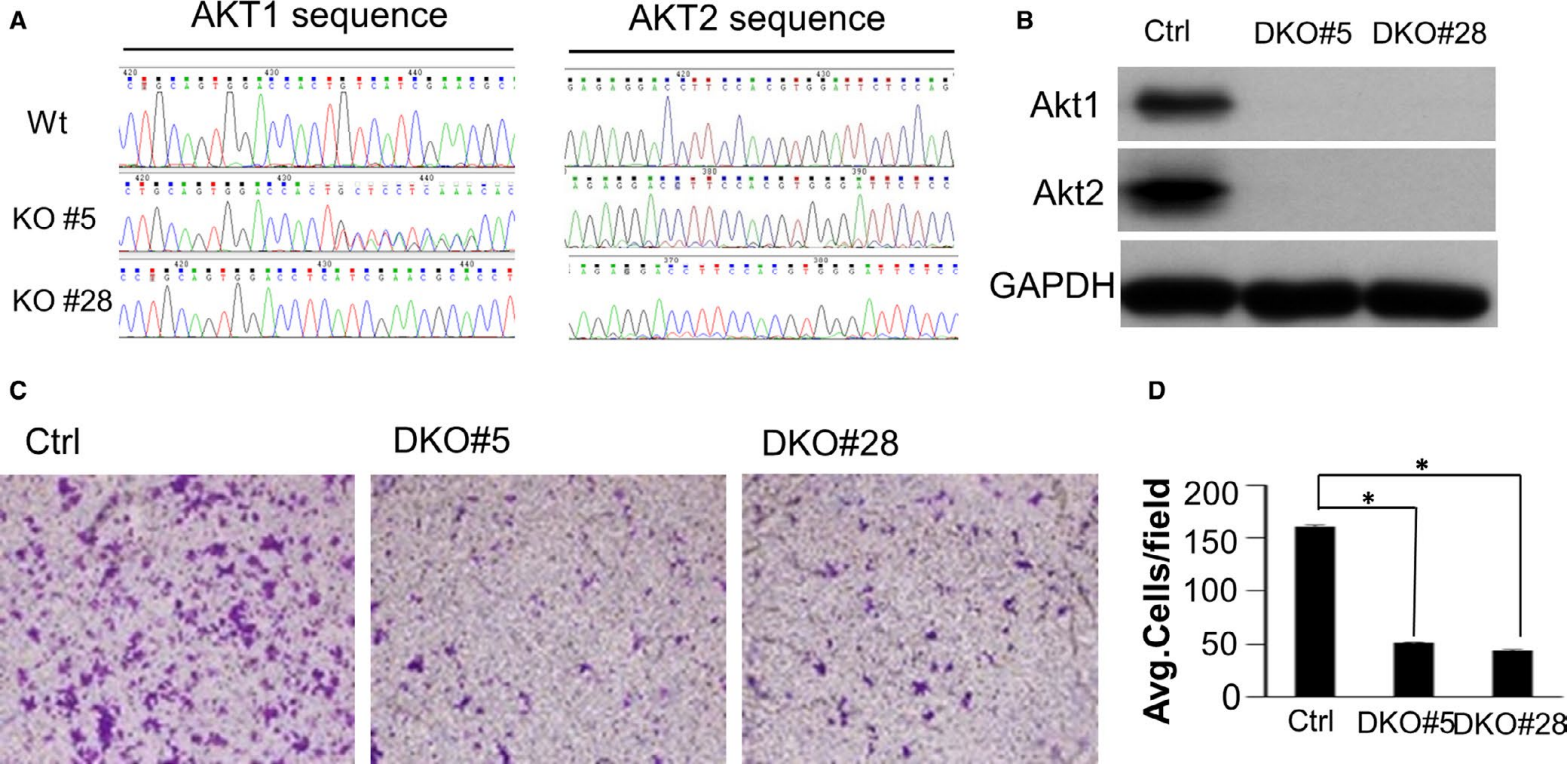
Effects of Akt1/2 knockout on metastasis in CaP CWR22rv1 cells in vitro. A, The sequencing analyses revealed that Akt1/2 was successfully knocked out in monoclonal cells (KO#5 and KO#28). B, Western blotting confirmed the successful knockout of both Akt1 and Akt2. C, Matrigel‐based invasion assay was performed in CWR22rv1/Akt1/2 DKO cells. D, The cell numbers per field were counted, and the results summarized in a bar graph. Error bars, SE of triplicate experiments. *P < .05

To interrogate the importance of Akt in CaP metastasis, our study directly targeted to exon 3 of the Akt1 and Akt2 genes (Figure S1A,B) and was performed on AR‐positive prostate cancer cell lines of CWR22Rv1. The sequencing analyses revealed that Akt1 and Akt2 were successfully knocked out from screening monoclonal cells of CWR22rv1 (Figure 1A). Western blotting further confirmed the successful knockout of both Akt1 and Akt2 (Figure 1B). To determine a potential role of Akt1/2 in CaP metastasis potential, we first examined whether knockout of Akt1/2 affects the invasiveness of CaP cells (Figure 1C). As expected, knockout of Akt1/2 resulted in twofold to fourfold decrease for invasiveness in two screening clones, as assessed by Boyden chamber‐mediated invasion assay (Figure 1D).

### 3.2 Effects of Akt1/2 knockout on metastasis in CaP CWR22rv1 cells in vivo

**FIGURE 2:**
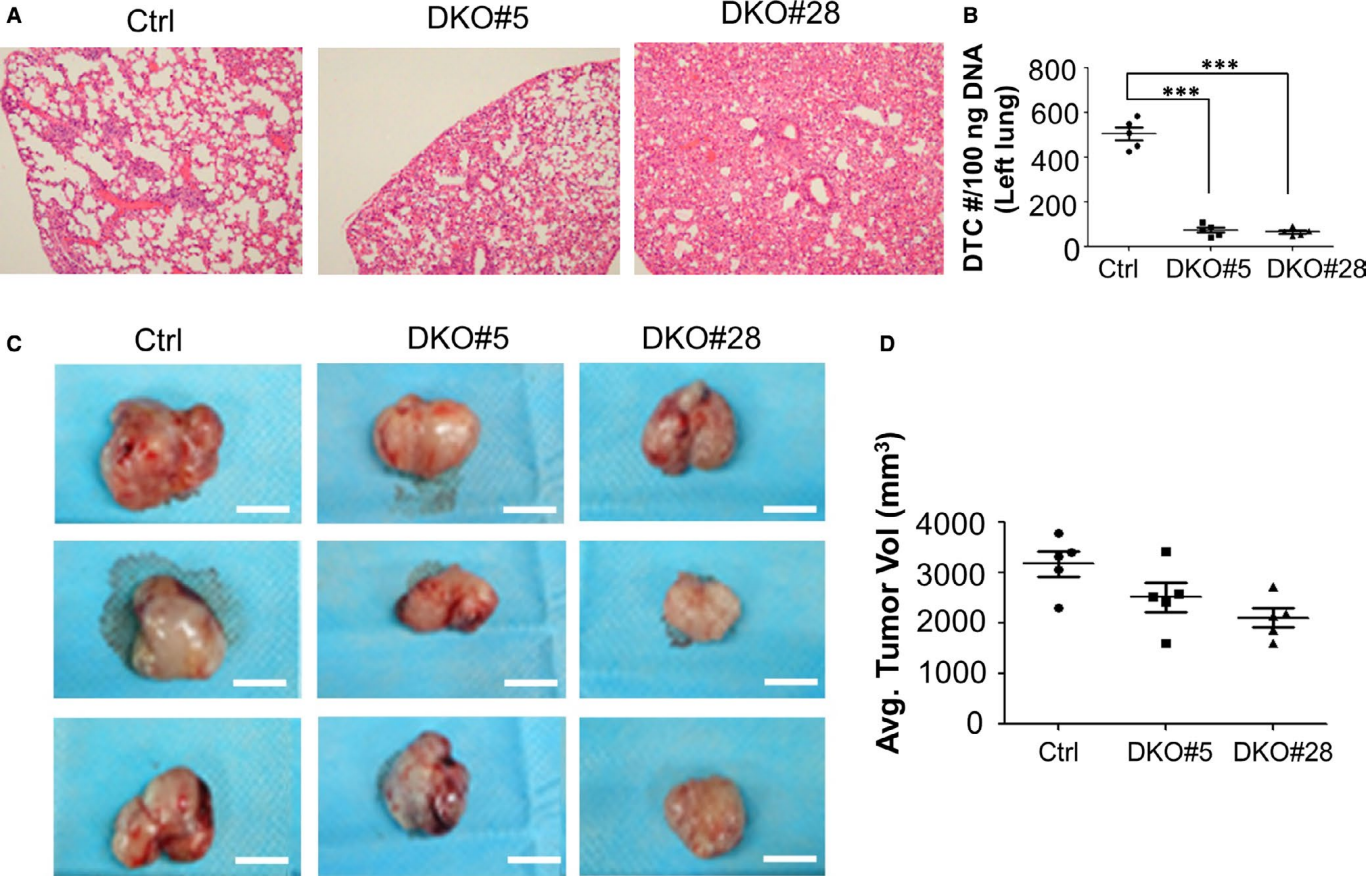
Subcutaneous xenograft CaP models were generated using CWR22rv1/Akt1/2 DKO cells. A, Representative images of the metastatic lung lesions. B, The existence of metastatic tumour cells in lungs was assessed by qPCR‐based detection of human Alu sequences, ***P < .001. C, Tumour growth was monitored. D, The average tumour volumes of primary tumours were calculated

We then tested whether Akt1/2 knockout (Akt1/2 DKO) could affect spontaneous metastasis in vivo in a subcutaneous xenograft model using CWR22rv1/Akt1/2 DKO cells and control cells in nude mice. When the primary tumours were grown in the mice exceeded 4 cm^3^, the mice were killed and the related organs were harvested and analysed for metastasis. None of the animals examined in this study were overt metastases in local draining lymph nodes, kidney and lung of the Akt1/2 DKO and control groups. Furthermore, micrometastases were evaluated with human Alu sequence by qPCR. Samples harvested from Akt1/2 DKO groups presented with significantly decreased incidence of metastases to lungs compared with the control group (*P* < .001) (Figure 2A,B). These observations confirmed that inhibition of Akt1/2 could repress CaP metastasis in vitro and in vivo. Akt1/2 DKO had marginal effects on primary tumour growth (Figure 2C,D).

### 3.3 Identification of candidate metastatic‐associated genes regulated by Akt1/2

**FIGURE 3:**
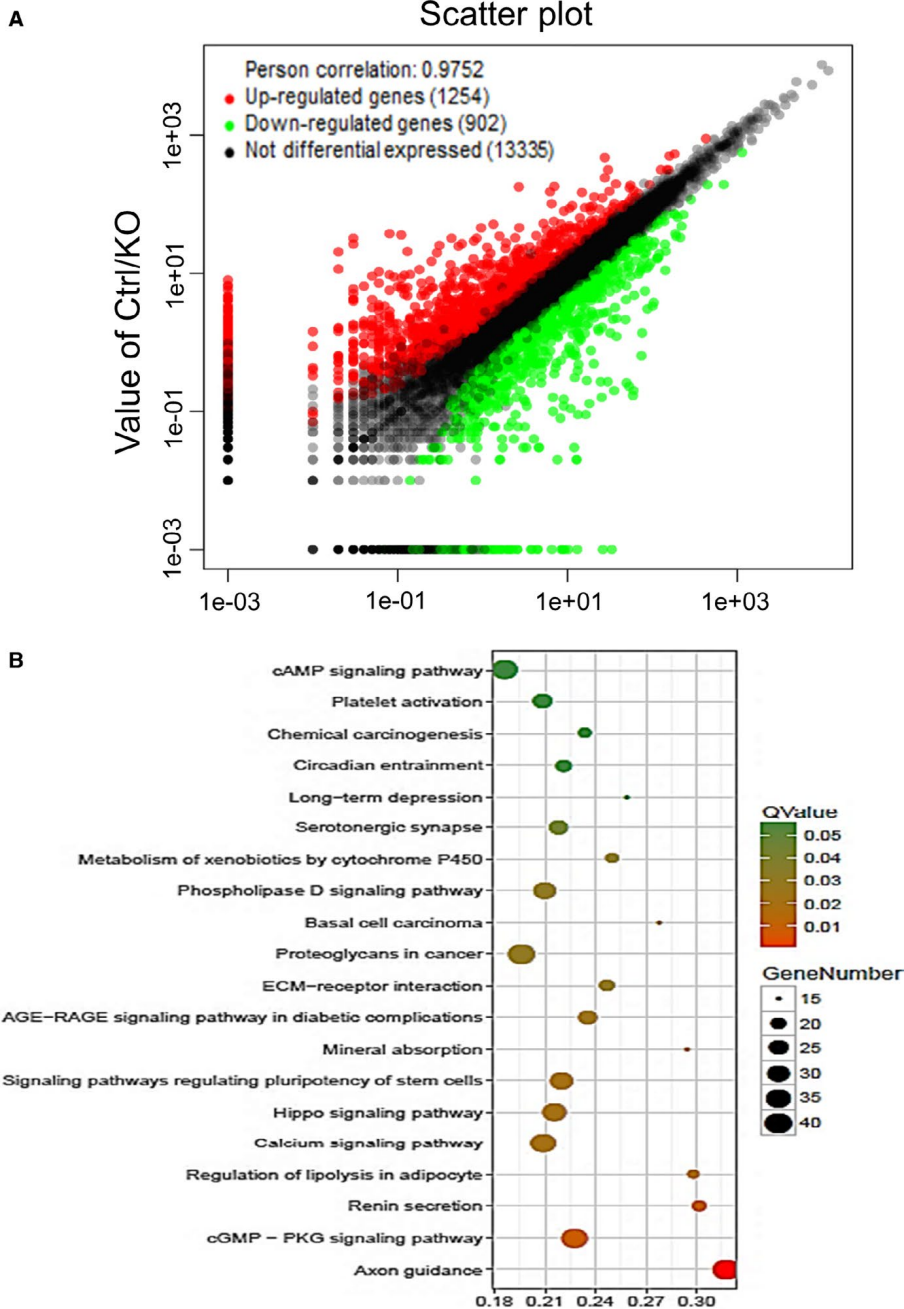
Identification of candidate metastatic‐associated genes regulated by Akt1/2. A, RNA sequencing was performed with CWR22rv1/Akt1/2 DKO cells and control cells. Scatter plot shows 2156 genes whose expression changed ≥ 1.5‐fold. B, Top 20 of Pathway Enrichment

In order to further explore the mechanism of Akt affecting CaP metastasis, we used RNA sequencing technology to analyse the effect of Akt1/2 knockout on transcriptional RNA of CWR22rv1 cells. This analysis identified 2156 genes whose expression changed ≥ 1.5‐fold (Figure 3A), which are involved in many aspects of cell biological functions including stem cells, metabolism and interaction between extracellular matrix and receptors (Figure 3B), etc

**FIGURE 4:**
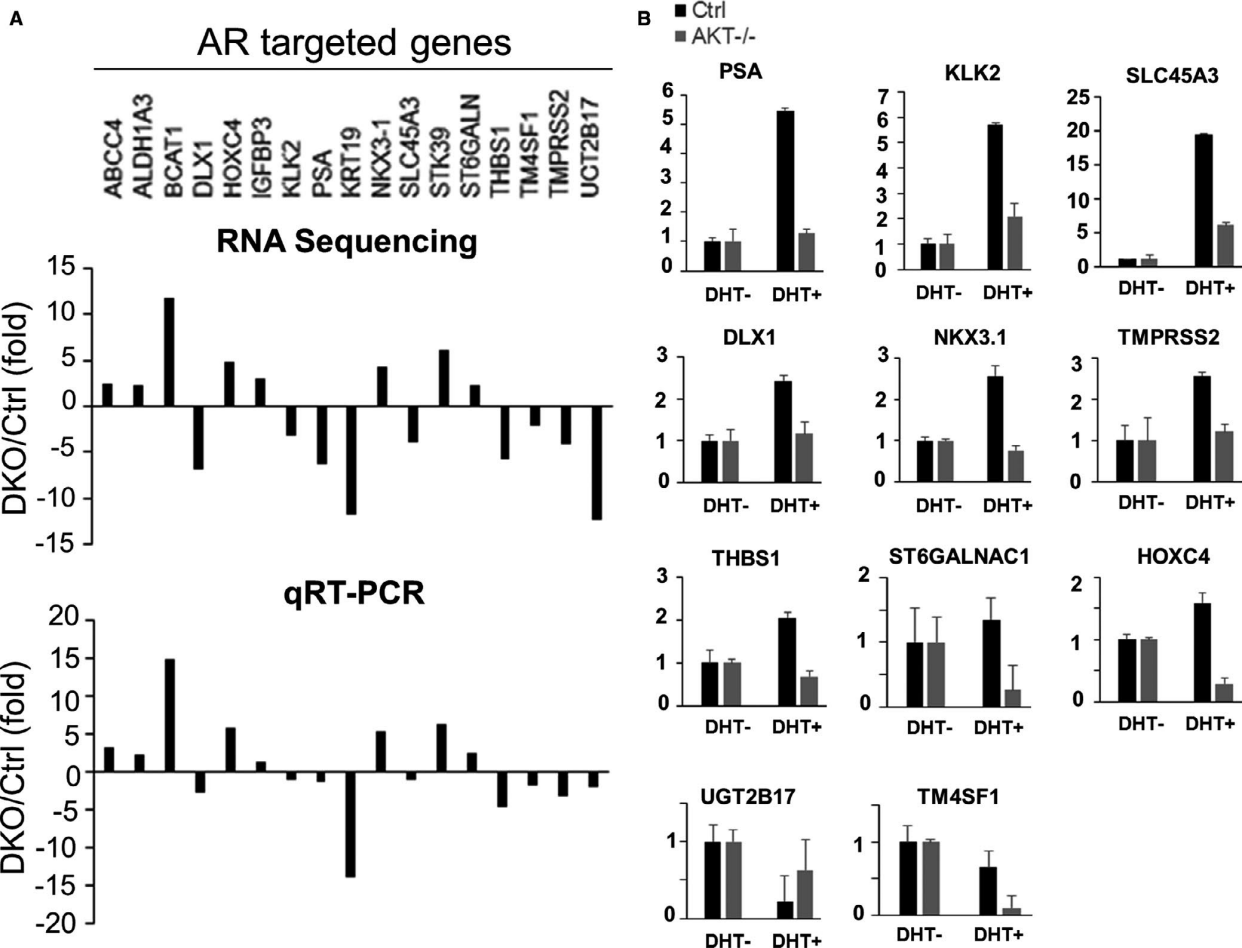
Androgen‐mediated expression changes in AR‐targeted genes were abolished in Akt1/2 DKO cells. A, The 17 genes are AR‐regulated genes. RNA sequence results were presented on the top panel. qRT‐PCR validation on the low panel. B, The expression of androgen‐mediated activation of these AR‐targeted genes was abolished in Akt1/2 DKO cells

Of these, bioinformatic analysis and literature search revealed a bunch of genes are AR‐regulated genes (Figure 4A) and involved in CaP metastasis‐associated processes such as NKX3.1,^10^ ST6GALNAC1,^11^ TM4SF1,^12^ UGT2B17^13^ and STK39.^14^ Furthermore, the expression of androgen‐mediated activation of these AR‐targeted genes was abolished in Akt1/2 DKO cells (Figure 4B). Not surprisingly, the interaction between PI3K/Akt and AR signalling pathways plays an important role in the development and metastasis of CaP,^15^ implying the Akt1/2 knockout‐reduced metastasis may be through AR signal pathway. However, knockout of Akt1/2 does not affect the level of AR, indicating that the metastasis suppression was not due to change on AR expression level.

### 3.4 FOXO activation decreased invasive ability of Akt1/2 DKO CWR22rv1 cells

**FIGURE 5:**
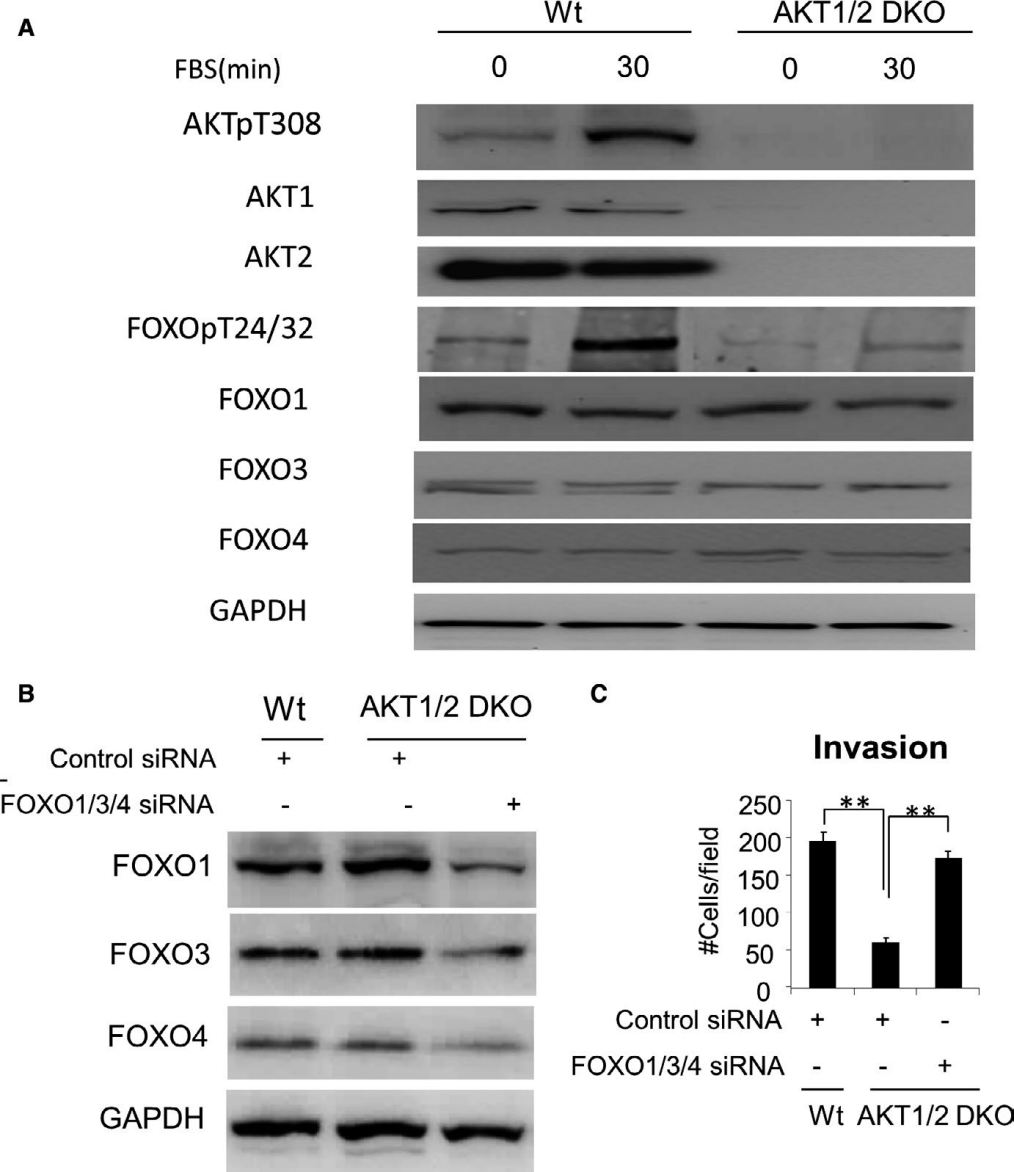
Knockout of Akt1/2 may interfere CWR22rv1 cell invasiveness through FOXOs. A, Effect of growth factor stimulation on FOXO phosphorylation in CWR22rv1/Akt1/2 DKO cells. B, Western blot confirms FOXO1/3/4 was successfully knockdown. C, Knockdown of FOXOs rescues decreased invasiveness, **P < .01

As we know, after Akt is activated by phosphorylation at Thr308, it regulates cell proliferation, survival, motility and metastasis through phosphorylating a host of downstream mediators, including GSK3β, FOXOs and mTOR. To evaluate the effects of gene disruption on known Akt‐signalling pathway, we starved cells of serum overnight and then stimulated them with foetal bovine serum (FBS). Striking changes in the levels of phosphorylated Akt and FOXOs were observed after FBS was added to control cells (Figure 5A).

To determine whether increased activity of FOXOs contributes to the decreased invasive capacity of Akt1/2 DKO CWR22rv1 cells, Akt1/2 DKO cells transfected with siRNA against FOXOs were tested for invasiveness. The results demonstrated that knockdown of FOXOs (Figure 5B) rescues decreased invasiveness (Figure 5C). However, quantitative PCR indicated that no significant changes detected on common target genes of FOXOs, such as p21 and p27 related to cell cycle, Bim and FasL genes related to apoptosis, LC3 related to autophagy and PEPCK and PDK‐4 related to metabolism (Data not shown), indicating that the metastasis suppression was not due to effects of FOXO transcription factor activity.

### 3.5 Knockout of Akt1/2 may interfere AR nuclear translocation through regulating FOXOs

**FIGURE 6:**
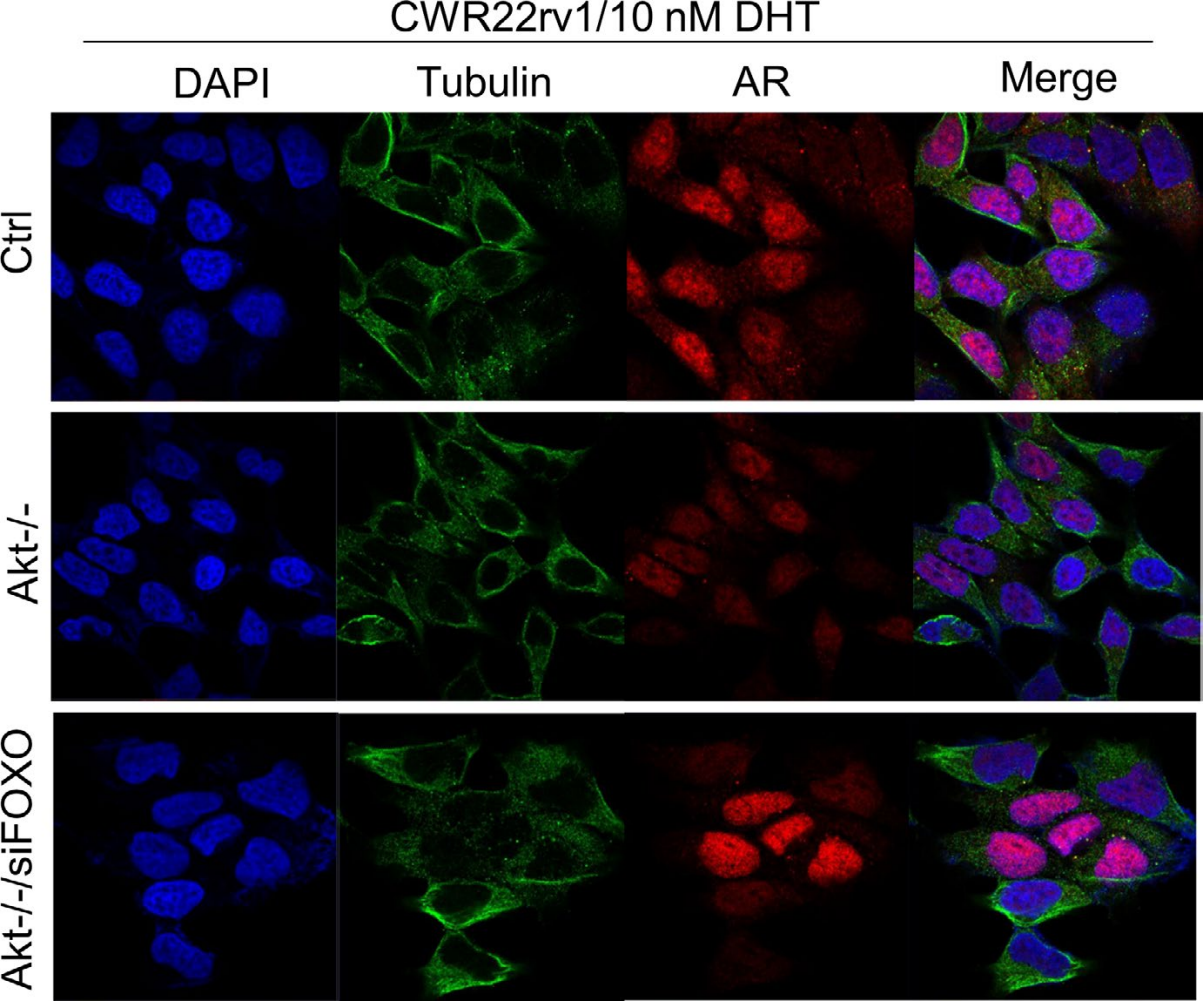
Knockout of Akt1/2 may interfere AR nuclear translocation through FOXOs. Cell immunofluorescence images of all nuclei (DAPI), tubulin and AR among negative control group, Akt1/2 DKO group and Akt1/2 DKO group with siFOXO

In addition to transcription factor activity, FOXO can affect the function of other proteins through protein‐protein interactions, such as FOXO1 can directly bind to AR and inhibit its activity to affect the progression of prostate cancer,^16^ suggesting FOXOs might affect Akt1/2 knockout‐reduced metastasis by interfering with AR nuclear translocation. Therefore, we investigated the effect of FOXO knockdown on AR nuclear translocation in Akt1/2 DKO CWR22rv1 cells. Following the treatment with DHT, AR largely translocated into the nucleus in control cells (Figure 6, upper), but knockout of Akt1/2 induced a marked reduction in AR nuclear accumulation (Figure 6, middle). As expected, knockdown of FOXOs rescued DHT‐stimulated AR nuclear translocation (Figure 6, lower), supporting our thought that knockout of Akt1/2 may interfere AR nuclear translocation through FOXOs.

## 4 DISCUSSION

In mammals, the FOXO family consists of four members (FOXO1, FOXO3, FOXO4, FOXO6), of which FOXO6 exists only in the brain, so the other three proteins are the major peripheral proteins.^17^ FOXO proteins are the important molecules downstream of Akt‐signalling pathway. Akt inhibits the function of FOXO by phosphorylating three sites of FOXO protein (Thr24, Ser256 and Ser319), transferring it from the nucleus to the cytoplasm and inactivating by binding to the 14‐3‐3 protein.^18^ FOXO family members are involved in the regulation of many cell functions, including apoptosis, DNA damage repair, cell cycle arrest and metastasis and are therefore considered tumour suppressor genes.^19^ We speculate that increased activity of FOXO proteins is responsible for the metastasis‐disruption effects of Akt1/2 DKO in CWR22rv1 cells. In cells of double knockout of Akt1/2 genes, no phosphorylated Akt proteins were observed as expected. Importantly, no phosphorylated FOXOs could be detected in these Akt1/2‐KO cells (Figure 6A). Other known constituents (GSK3β and mTOR) of the Akt pathway were not affected to the same degree (Data not shown). Our findings are consistent with those of Dr Vogelstein's findings^20^ that enhanced FOXO protein activity was correlated with reduced liver metastasis after simultaneous knockout of Akt1/2 in human colon cancer cells.

At present, the development of anti‐tumour drugs is mainly focused on inhibiting major tumour‐related genes, such as AR and Akt. Many inhibitors targeting these two genes have been developed and entered clinical validation,^21^ but the clinical results are impressive disappointed, most patients will still progress to CRPC and eventually die of metastasis.^22^ It is reasonable when the major tumour‐related genes are blocked, they suppress the malignant aspects of tumour cells, but simultaneously, the normal functions of these genes, such as the basic physiological functions of maintaining normal tissue balance, are also destroyed, so that these measures are successful in the short term, but the end result is not ideal. Hence, new and innovative treatment approaches are needed. Recently, with the deepening of the research on the mechanism of AR to regulate the progression of CaP, instead of inhibiting AR, new therapeutic research focuses on AR‐interacting proteins, such as Geldanamycin, an inhibitor of AR chaperone HSP90, to accelerate the degradation of AR, and SRC inhibitor of AR co‐regulator agent Sangivamycin blocks the transcriptional activity of AR, etc These new approaches have brought dawn to the treatment of prostate cancer.^23^

In short, it suggests that some downstream regulatory factors in the AKT and AR interaction network play a vital role in prostate cancer metastasis and are potential targeting molecules for prostate cancer metastasis treatment.

## CONFLICT OF INTEREST

The authors declare that there are no conflicts of interest.

## AUTHOR CONTRIBUTION

**Bing Su:** Conceptualization (equal); Writing‐original draft (lead). **Lijuan Zhang:** Investigation (supporting); Methodology (supporting). **Wenfang Zhuang:** Data curation (lead). **Wei Zhang:** Conceptualization (supporting); Writing‐original draft (supporting). **Xiaofan Chen:** Investigation (lead); Methodology (lead).

## Supporting information

Fig S1

Table S1

## Data Availability

All raw and processed sequencing data have been submitted to the NCBI Gene Expression Omnibus (GEO; http://www.ncbi.nlm.nih.gov/geo/) under accession number GSE162743.
