# Supplementary figures and images for: Knockout of Akt1/2 suppresses the metastasis of human prostate cancer cells CWR22rv1 in vitro and in vivo
Source: J Cell Mol Med. 2020 Dec 29;25(3):1546–53. doi: 10.1111/jcmm.16246 (PMC7875906; doi:10.1111/jcmm.16246)

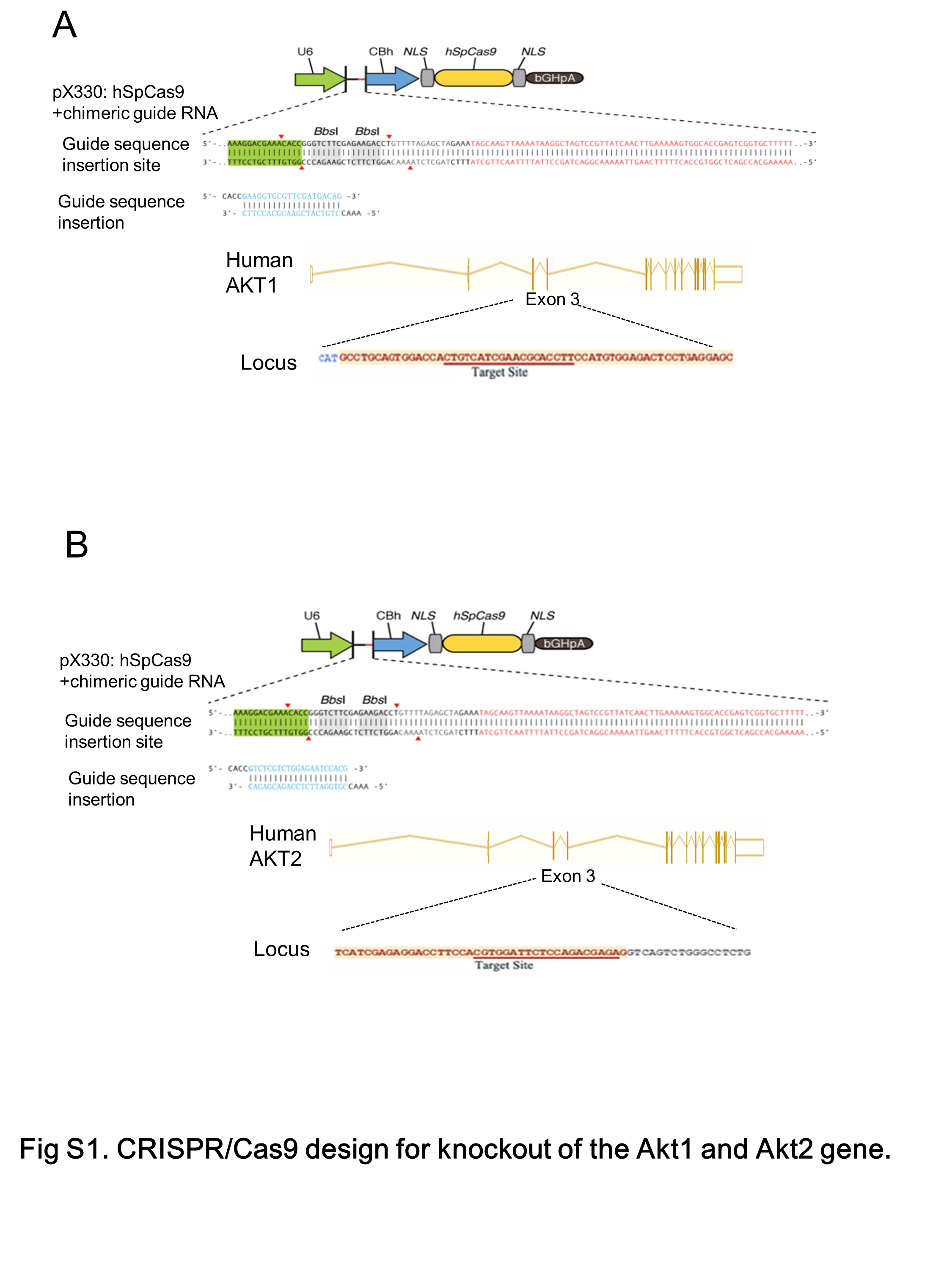

Supplement: Supplementary file 1 — Fig S1 [file JCMM-25-1546-s001.TIF]
