# Supplementary material for: Knockout of Akt1/2 suppresses the metastasis of human prostate cancer cells CWR22rv1 in vitro and in vivo
Source: J Cell Mol Med. 2020 Dec 29;25(3):1546–53. doi: 10.1111/jcmm.16246 (PMC7875906; doi:10.1111/jcmm.16246)
Supplement: Supplementary file 2 — Table S1 [file JCMM-25-1546-s002.docx]

**Table S1. List of primers used in this study.**

| Primer Name | Sequence |
| --- | --- |
| ABCC4 | ATTGAGAGGGTGTCAGAGGC |
|  | CAATTCGCCAGGTCTGACAG |
| ALDH1A3 | AAGAGGAGATTTTCGGGCCA |
|  | GCAGTTGATCCAGACCGTTC |
| BCAT1 | CCAGGCCTTGCAGAAATTGT |
|  | TCACCGAGCACTCTGACATT |
| IGFBP3 | TCTGATCCCAAGTTCCACCC |
|  | TCCATTTCTCTACGGCAGGG |
| PSA | CGGATGCTGTGAAGGTCATG |
|  | CTTCTGAGGGTGAACTTGCG |
| KLK2 | GTTTGTGGGGTGCAGAGATG |
|  | CCTCCCCTTCTTTCTGCTCA |
| NKX3.1 | TCTGCAACTCCATCCTCCTG |
|  | CAGAGAGAGCCTTGGCCATA |
| DLX1 | TGGAGAGTTTGGAGCCGAAT |
|  | CGCTCCCTCTTTCTACCCAA |
| KRT19 | CTGCGGGACAAGATTCTTGG |
|  | GTAGGCCAGCTCTTCCTTCA |
| SLC45A3 | CACTCATGACCTTCACGCTG  TGGCCAAATAGACTGCTCGA |
| STK39 | CTGGGTGAGGATGGTTCAGT |
|  | ACTCCACATGTCAGCCTTGA |
| TMPRSS2 | TCACACCAGCCATGATCTGT |
|  | CATTCCCGTACACTCCTGGT |
| THBS1 | GATGCCTGCTTCAACCACAA |
|  | ACATGGGGTCGCTATAGTGG |
| ST6GALNAC1 | GAACACTTTGCACCACCCTT |
|  | GACTGTCTATCTCCTGGCCC |
| HOXC4 | TCCTCTCCCTCCCACTGTTA |
|  | AAGCCAGACCATCACACCTT |
| UGT2B17 | TGAAAATGTTCGATAGATGGACATATAGTA |
|  | GACATCAAATTTTGACTCTTGTAGTTTTC |
| TM4SF1 | ATCGGACATTCTCTGGTGGG |
|  | CCACAGTTTTCATGGCCACA |
